# Multidimensional Latent Semantic Networks for Text Humor Recognition

**DOI:** 10.3390/s22155509

**Published:** 2022-07-23

**Authors:** Siqi Xiong, Rongbo Wang, Xiaoxi Huang, Zhiqun Chen

**Affiliations:** 1 College of Computer Science and Technology, Hangzhou Dianzi University, Hangzhou 310018, China; xsqzjyy@163.com (S.X.); huangxx@hdu.edu.cn (X.H.); chenzq@hdu.edu.cn (Z.C.); 2 Institute of Cognitive and Intelligent Computing, Hangzhou Dianzi University, Hangzhou 310018, China

**Keywords:** humor recognition, humorous semantic features, discourse understanding, human-computer interaction, deep learning

## Abstract

Humor is a special human expression style, an important “lubricant” for daily communication for people; people can convey emotional messages that are not easily expressed through humor. At present, artificial intelligence is one of the popular research domains; “discourse understanding” is also an important research direction, and how to make computers recognize and understand humorous expressions similar to humans has become one of the popular research domains for natural language processing researchers. In this paper, a humor recognition model (MLSN) based on current humor theory and popular deep learning techniques is proposed for the humor recognition task. The model automatically identifies whether a sentence contains humor expression by capturing the inconsistency, phonetic features, and ambiguity of a joke as semantic features. The model was experimented on three publicly available wisecrack datasets and compared with state-of-the-art language models, and the results demonstrate that the proposed model has better humor recognition accuracy and can contribute to the research on discourse understanding.

## 1. Introduction

Humor is an important flavoring agent in human life and communication, often easing tensions and providing an expression way for socially unacceptable feelings, behaviors, and impulses [1]. However, text is an important medium for conveying humor, and as the field of artificial intelligence (AI) places more and more focus on the study of text, the exploration of deeper semantic directions of text is becoming increasingly popular, one of which is computational humor. The computational humor is based on humor theory in linguistics to reveal the mechanisms of human humor and to build a linguistic model with humor cognitive functions for providing a basis for deeper human–computer interaction [2]. For practical applications, if computers can recognize humor expression, then true intentions of authors can be understood more accurately for making human–computer interaction more interesting and engaging.

Jokes, generally short but simple and clever, are an important style for humor expression. Identifying whether a joke is humorous or not is an important task in computational humor. Humor recognition is often referred to as a binary classification task that detects whether a text is humorous or not. As there are different forms of humorous texts, such as dialogues, stories, and short jokes (witticisms), we cannot use a unified model to identify them. Therefore, in this paper, the humor recognition task focuses on short jokes because they can use fewer words to express humor. They have no complex semantic structures and longer contexts of long jokes, they have no complex interpersonal relationships of dialogues, and they usually generate humor through puns, rhymes, and inconsistencies in semantics. Normally, the punch line of a joke is brought in by a few wise words at the end to create inconsistencies and thus make the text humorous [3]. Humor usually also has the ambiguity and phonetic features mentioned.

In recent years, there has been a growing emphasis on humor recognition, mostly in the context of textual humor recognition; many of the assessment tasks also have humor-related tasks [4,5] identifying humor by incorporating theoretical understanding of the linguistics of humor and extracting humorous features [6,7]. Many linguistic features are proposed for humor recognition, such as incongruity structure, ambiguity theory, interpersonal effect, phonetic style, and so on. However, manually constructing the features needed for humor recognition is difficult and laborious. With the development of deep-learning-based approaches, a number of methods have been proposed in recent work; for example, Kumar et al. [8] propose a combination of convolutional neural networks (CNN) and long short-term memory (LSTM), with the addition of a highway to enhance performance; Weller et al. [9] proposed the use of the transformer architecture to take advantage of its learning from the context of sentences; Lu Ren et al. [10] proposed to combine humor recognition and pun recognition, training the two tasks jointly, thus enhancing performance. There is also humor recognition through multimodal means [11,12]. Unlike these works, we propose a deep learning method approach based on humor linguistics to capture inconsistency features, phonetic features, and ambiguity features in humor, introducing the incongruity of humor caused by semantic inconsistency, lexical, and ambiguity.

In this paper, we propose a model to implement these methods that analyzes multiple characteristics of humor and extracts multiple features of a joke in order to determine whether a sentence contains humor or not. The contributions of the proposed are listed as follows:

- In order to identify the inconsistency, fuzzy features, and phonetic features of humor, a model is designed which is able to extract the fragment embeddings of jokes via RoBERTa word embeddings and CNNs, semantic inconsistency features using Glove word embeddings, CNNs, and transformer encoders, phonetic features in jokes via Carnegie Mellon University’s pronunciation dictionary (CMU), and CNNs and fuzzy features using WordNet, Bi-LSTM, and attention mechanisms.
- Compared to previous work, this paper uses a new extraction approach for the three features proposed above. Different from exploring the relationship between words, in terms of extracting text inconsistencies, we no longer extract inconsistency features between words and words, but inconsistency features between snippets; obtain the phonetic features of sentences by converting words into phonetic sequences; and extraction of fuzzy features by exploring the relationship between words and synonyms.
- The model achieves superior results compared to the state-of-the-art models available on three datasets, pun-of-the-day [13], 200K-Oneliners [14], and SemEval-2021 Task-7 [15], respectively.

## 2. Related Work

### 2.1. Domestic and International Theoretical Research on Humor

The definition of humor in Cambridge Dictionary is the ability to be amused by something seen, heard, or thought about, sometimes causing you to smile or laugh, or the quality in something that causes such amusement. At present, there are comprehensive studies on humor, namely, three main theories: the superiority theory from a social behavioral perspective, the release theory from a psychoanalytical perspective, and the perverse theory from a psycho-cognitive perspective [16].

The study of humor theories provides important guidance on semantic and pragmatic rules for humor recognition, and builds the theoretical foundation for extracting humor features for humor recognition in this paper.

### 2.2. Relevant Studies in Text Humor Recognition

Naturally, there are many studies on humor detection in the form of one-liners. Feature engineering approaches are highly popular, exploiting a diverse range of features such as stylistic [17], distribution of part of speech [18], and affective dimensions [19]. Purandare and Litman [20] analyzed acoustic–prosodic and linguistic features to automatically recognize humor during spoken conversations.

In recent years, humor recognition has been a very challenging task in natural language processing, making many researchers work for it. Mihalcea and Strapparava [3] extracted head rhymes, antonyms, and adult slang features for humor recognition. Mihalcea et al. [21]. artificially divided the witticisms into two parts: the “set up” and the “punchline”, and used the semantic conflicts between them as the basic features of humor recognition, using semantic similarity calculation and some humorous linguistic features for humor recognition. Zhang et al. [19] designed up to 50 humorous features in 5 major categories based on the Twitter corpus. Yang et al. [13] constructed four types of features for humor recognition, including inconsistency, ambiguity, interpersonal effects, and speech style.

In deep learning, humor recognition has also achieved excellent results. Bertero and Fung [22] combined word-level and audio-frame-level features and used RNNs and CNNs to predict humorous discourse. De Oliveira and Rodrigo [23] also applied LSTM to detect humor from reviews in the Yelp dataset and achieved satisfactory results. With the development of pretrained language models in natural language processing, some excellent pretrained language models have been applied for humor recognition tasks. Mao and Liu [24] proposed a humor recognition method based on BERT. Ma et al. [25] proposed an enhanced inference Bert (EI-Bert) based on different feature sentence pairs for humor recognition. At the same time, humorous datasets are also very difficult to obtain. Wu J. et al. [26] constructed a novel multimodal dataset named MUMOR for humor recognition, and Blinov V. et al. [27] collected a dataset of jokes and funny dialogues in Russian from various online resources and complemented them carefully with unfunny texts with similar lexical properties. The dataset comprises more than 300,000 short texts, which is significantly larger than any previous humor-related corpus.

However, the above research results only extracted one or two features without combining them; at the same time, researchers focused only on word-to-word relationships while ignoring snippet-to-snippet relationships. Therefore, this paper proposes a humor recognition model that incorporates text fragment inconsistency, phonetic features, and fuzzy features.

## 3. Analysis of Humor Features

Based on previous research on humor theories, this paper proposes three potential semantic features about humor, namely, inconsistency feature, speech feature, and fuzzy feature, for which the identical three features are designed and implemented in this paper, and then these features are combined for humor recognition.

### 3.1. Inconsistent Feature

The semantic scripting theory of humor [19] stated that inconsistency was one of the important causes of humor. The production of humor often depends on some incongruous, contradictory, or obviously contradictory combination [28]. That is, there is no direct relationship between one idea and other ideas. For example, “A clean desk is a sign of a cluttered desk drawer”. In this example, there is a certain inconsistency between “clean desk” and “cluttered desk drawer”, thus generating a certain humorous effect. Since it is difficult to directly confirm the inconsistency, semantic analysis is required to simplify this work. In recent years, with the development of deep learning applications, it has achieved remarkable results in text semantic representation [29].

### 3.2. Fuzzy Feature

The relevance theory of humor [30] mainly explores and analyzes humor from a common phenomenon in natural language, namely, ambiguity. When a word without ambiguous meanings has multiple meanings [31], it usually becomes an important sign of producing humor. The main reason for ambiguity is that some words have superficial meanings, but they are “forced” to produce a deeper and more obscure meaning due to the constraints of context. For example, “Did you hear about the guy whose whole left side was cut off? He’s all right now”. In this sentence, besides the meaning of “on the right”, “right” has another meaning of “recovery” when combined with “all”. In order to solve similar problems, WordNet is usually introduced, and the farthest and nearest paths of the meaning contained in it are compared, so as to realize the discovery of ambiguity features. Based on this point, the humor caused by the ambiguity of words or phrases is identified.

### 3.3. Phonetic Feature

Other theories of humor also suggest that phonological properties are also important in generating humor [32]. Phonological properties make texts that are not originally humorous or funny. Jokes usually depend on being read aloud to produce comic effects through head rhymes, superlatives, rhymes, etc. Similar methods are often found in newspaper headlines, hymns, and jingles. Head rhyming chains usually refer to two or more words that begin with the identical pronunciation, while rhyming refers to words that end a sentence with the identical syllable between them. In order to extract features of pronunciation types, the Carnegie Mellon University Pronunciation Dictionary (CMU Pronouncing Dictionary) can be applied to implement humorous recognition of pronunciation categories.

## 4. Methods

**Figure 1:**
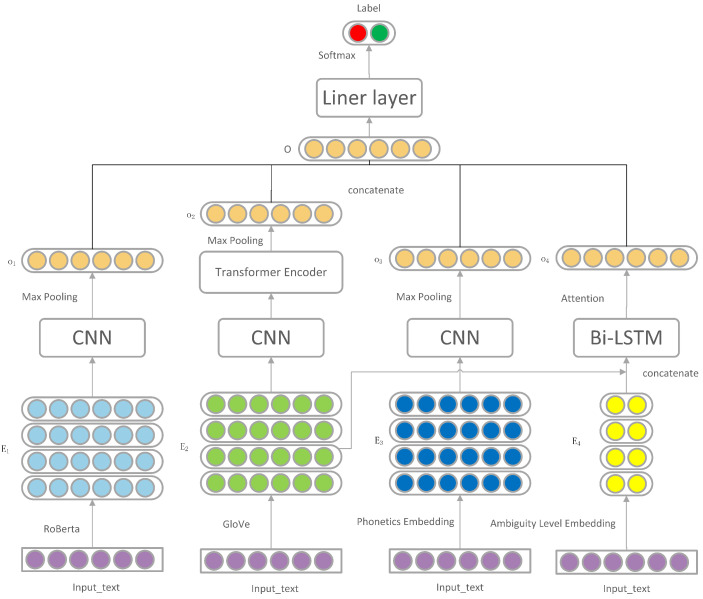
The framework diagram of the model consists of four parts: snippet embedding module, snippet semantic inconsistency module, voice feature module, and ambiguity module.

In this section, the model is introduced in detail. The model improves the humor recognition through the three dimensions of joke inconsistency, phonetic features, and fuzzy features. The framework of this model is shown in Figure 1.

The framework of the model consists of four main components:

1. The snippet embedding module that uses RoBERTa and convolutional neural networks for joke snippet extraction embedding;
2. Semantic inconsistency extraction module applies convolutional neural network and transformer encoder to extract the semantic inconsistency of jokes;
3. Phonetic feature module to determine humor by convolutional neural network speech embedding;
4. Using Bi-LSTM and attention mechanism for textual-ambiguity-related ambiguity modules.

The details of the proposed model in this paper are presented in the following sections.

### 4.1. Snippet Embedding Module

Previous studies on humor recognition about inconsistency focused only on the inconsistency between words. Cao [33] proposed the relationship between fragments and improved the model on the above basis. RoBERTa was used to embed words, and convolutional neural network (CNN) was used to intercept fragments to give sentence representation.

#### 4.1.1. Word Embedding

RoBERTa is an upgraded version of BERT [34,35] and has superior performance compared to BERT. RoBERTa improves upon BERT as follows:

1. Longer training time, larger batch size, more training data;
2. Removes the next predict loss;
3. Longer training sequences;
4. Dynamic adjustment of the masking mechanism.

In this paper, RoBERTa word embedding is chosen as the word embedding of the snippet embedding module, and after word embedding, it can be represented as $E_{1}\in R^{N\times e}$, where N is the sequence length and e is the word embedding dimension.

#### 4.1.2. Convolutional Layer

Since the final result of this module is a sentence snippet embedding, the jokes are required to be divided, and the convolutional neural network is just adapted to this module, which can be applied to grasp contextual local features by implementing convolutional operations between a convolutional kernel and a series of word embeddings. From this, the convolution filter size is set to $f\in R^{l\times e}$ and the step length equals 1. The input is operated by the convolution layer to obtain $C\in R^{\left(l-N+1\right)\times e}$, and *C* is a set of vectors *C*={$c_{1},c_{2},c_{3},\ldots ,c_{l-N+1}$}. The formula is as follows:

(1) $$c_{i}=\sum E_{i,e}⊗f_{N,e},$$

Finally, the max pooling layer is passed until $o_{1}\in R^{n}$, and n is the convolutional layer output channel size.

### 4.2. Semantic Inconsistency Extraction Module

Inconsistency is widely considered as a humorous feature, and inconsistencies between semantics are not necessarily inconsistencies between words, but also between snippets. Sentence inconsistencies can be considered to some extent as oppositions or contradictions between semantic blocks of sentences.

#### 4.2.1. Word Embedding

This module applies GloVe [36] for word embedding to obtain a sentence-level representation, which is fixed during training. The tokens of all unknown words are replaced with “<unk>”. To obtain a uniform sentence length, each edited sentence is either truncated or filled with “<pad>”. Embeddings for unknown word symbols and padded token symbols are initialized with zero vectors and random vectors, respectively. Finally, the word embedding is denoted as $E_{2}\in R^{N\times b}$; N is the sequence length and b is the embedding dimension.

#### 4.2.2. Sentence Slicing and Capturing Semantics

Sentences are divided into many parts, as described in Section 4.1.2, the semantic block vector is obtained by dividing, and then the semantic inconsistency information of the text is obtained by the transformer encoder [37]. The attention vector $a_{i}$ from a self-attentive structure is computed as follows:

(2) $$a_{i}=\sum_{j=1}^{l-m}softmax\left(\frac{Q_{i}K_{j}^{T}}{\sqrt{e_{i}}}\right)V_{j}$$

where $e_{i}$ is the dimension of $Q_{i}$ and *j* is the number of sentence snippets. *Q*, *K*, and *V* represent three different types of encoded sentence fragment representations from GloVe. They can be calculated as follows:

(3) $$S_{Q,K,V}=W_{Q,K,V}^{i}*c^{i}+b_{Q,K,V}^{i}$$

where $c^{i}$ is the *i*-th segment representation, $W_{Q,K,V}^{i}$ is the corresponding projection matrix, and $b_{Q,K,V}^{i}$ is the bias term. Finally, the output $o_{2}\in R^{n}$ is obtained, where n is the output channel size of the convolutional layer.

### 4.3. Phonetic Feature Module

For a joke, in addition to inconsistency, phonetic feature is also an important feature for identifying humor. It makes a sentence humorous that is originally semantically not humorous, so we cannot ignore it.

#### 4.3.1. Phonetic Embedding

In this paper, we use Carnegie Mellon University (CMU)’s pronunciation dictionary to obtain the speech representation of jokes $E_{3}\in R^{N\times k}$, for example, “however” can be decomposed into “HH AW2 EH1 V ER0”.

#### 4.3.2. Convolutional Layer

Then, we use the convolutional neural network to obtain the local features of the speech sequence and obtain the output, $o_{3}\in R^{n}$.

### 4.4. Ambiguity Module

Ambiguity, the disambiguation of words that have multiple meanings, is an important part of many humorous jokes. That is, humor is produced by exploiting semantic and pragmatic ambiguities, which are closely related to the different meanings that a word, phrase, or sentence may have. Therefore, this paper pays special attention to the ambiguity of words, and explores the possible impact of these ambiguous words on humor recognization.

#### 4.4.1. Word Ambiguity Embedding

For the ambiguity of each word, this paper adopts a scoring mechanism according to the number of ambiguities in each word. For an input sentence, S={$w_{1}$,$w_{2}$,$w_{3}$,…,$w_{N}$}, using WordNet as an external resource, it calculates the number of synonyms for each $w_{i}$ and classifies them according to the number of synonyms. In this paper, four levels are taken. Stop words are one level independently, and each level is equal to a different weights, which is initialized by a random vector, where N is the length of a sentence, and finally the ambiguous word level sequence T={$t_{1}$,$t_{2}$,$t_{3}$,…,$t_{N}$} of the sentence is obtained; $t_{i}\in R^{N}$, k is the dimension.

#### 4.4.2. Word Embedding

To concatenate the word embedding vector obtained in Section 4.2.1 with the word ambiguity embedding vector obtained in Section 4.4.1, the output vector is $E_{4}\in R^{\left(k+b\right)\times N}$.

#### 4.4.3. Semantic Understanding Layer

Bi-directional long short-term memory (Bi-LSTM) [38] is added to the embedding layer to model the temporal interaction between humorous text words. The Bi-LSTM consists of a forward LSTM [39] and a backward LSTM to represent the contextual representation in two opposite directions, which avoids the vanishing gradient and scaling problems by learning the long-term dependencies of the text. The design of Bi-LSTM includes three gates and one unit for modeling semantic and contextual relations. This module applies the output of the embedding layer as input information, and then the update process of each forward LSTM network of Bi-LSTM is formulated as follows:

(4) $$i_{t}=\sigma \left(\vec{W_{i}}•\left[\vec{h_{t-1}},\vec{E_{4,t}}\right]+\vec{b_{i}}\right)$$

(5) $$f_{t}=\sigma \left(\vec{W_{f}}•\left[\vec{h_{t-1}},\vec{E_{4,t}}\right]+\vec{b_{f}}\right)$$

(6) $$o_{t}=\sigma \left(\vec{W_{o}}•\left[\vec{h_{t-1}},\vec{E_{4,t}}\right]+\vec{b_{o}}\right)\;$$

(7) $$g_{t}=\tanh\left(\vec{W_{c}}•\left[\vec{h_{t-1}},\vec{E_{4,t}}\right]+\vec{b_{c}}\right)$$

(8) $${\vec{c}}_{t}=f_{t}*\vec{c_{t-1}}+i_{t}*g_{t}$$

(9) $$\vec{h_{t}}=o_{t}⊙\tanh\left(\vec{c_{t}}\right)$$

(10) $$h_{t}=\left[\vec{h_{t}},\overset{\leftarrow }{h_{t}}\right]$$

where *t* is the step size, $f_{t}$ is the forgetting gate, $o_{t}$ is the output gate, $c_{t}$ is the storage cell, and σ is the sigmoid activation function. $W_{i}$, $W_{f}$, $W_{o}$, and $W_{c}$ are learned weights; $b_{i}$, $b_{f}$, $b_{o}$, and $b_{c}$ are bias values; $\vec{h_{t}}$ is the output of the forward LSTM, which together with the output of the backward LSTM ($\overset{\leftarrow }{h_{t}}$) forms the vector $h_{t}$.

#### 4.4.4. Attention Layer

In order to obtain the attention signal of humorous sentences according to the given ambiguity level of each word, the proposed model designs an attention mechanism [40]. The forward neural network is applied to calculate the semantic relevance of each word and its ambiguity level; the formula is as follows:

(11) $$g_{am}=ReLU\left(W_{am}h_{t}+b_{am}\right)$$

(12) $$\alpha_{am}=softmax\left(\omega^{t}g_{am}\right)$$

(13) $$v_{am}=H\alpha_{am}^{T}$$

where $g_{am}\in R^{\left(d+k\right)\times N}$, $\alpha_{am}\in R^{N}$, $\omega \in R^{d+k}$, and $v_{am}\in R^{d\times m}$; $\alpha_{am}$ is the importance weight normalized by the softmax function; $v_{am}$ is the context vector; and H is the output of the Bi-LSTM network.

This design is able to assign an appropriate word importance score by computing the semantic relatedness between a word and its ambiguity score. The final output $o_{4}\in R^{n}$.

### 4.5. Prediction Layer

The output vectors obtained by the previous four modules are connected, and finally the complete prediction layer input vector $O\in R^{4n}$ is obtained, and this input vector is injected into the prediction layer. The formula is as follows:

(14) $$\nu =ReLU\left(W_{p}\left[o_{1},o_{2},o_{3},o_{4}\right]+b_{p}\right)$$

where $W_{p}\in R^{4n\times 2}$ is the weight, $b_{p}$ is the bias value, $v\in R^{2}$ is result, and the obtained vector goes through the softmax classification layer to obtain the predicted value:

(15) $$\hat{y}=softmax\left(W_{f}\nu +b_{f}\right)$$

where $W_{f}$ is the weighting matrix, $b_{f}$ is the bias value, and $\hat{y}$ is the predicted label of the proposed model.

We apply cross-entropy loss in MLSN model. The loss is given by:

(16) $$loss=-\sum_{i=1}^{M}\sum_{j=1}^{N}y_{i}^{j}\log{\hat{y}}_{i}^{j}+\lambda {∥\theta ∥}^{2}$$

Here, *M* is the total number of all texts, and *N* is the number of classes. *y* is the true label of the text, and *y* denotes the predicted label of our model. *i* is the index of the text, *j* is the index of class, λ is the regularization parameter, and θ means all of the parameters in the model. The goal of the training is to minimize the loss function.

## 5. Experiment and Result Analysis

This section mainly introduces the experimental data and various baselines, compares the performance of each classifier, and concludes that the performance of the proposed model in this paper is better than the current state-of-the-art models.

### 5.1. Experimental Data and Evaluation Criteria

Pun-Of-The-Day (Puns): The dataset was collected by Yang et al., with humorous texts from websites of the same name, and non-humorous texts from AP News, New York Times, Yahoo News, and Proverbs. To avoid the classification problem caused by data imbalance, the numbers of negative and positive cases are basically the same distribution, and the total number of negative samples is 2403.

200K-Oneliners: The dataset contains 200k labeled short texts, evenly distributed between humorous and non-humorous. It is much larger than previous datasets and includes texts with similar textual features. The correlation between the number of characters and the target is not significant (+0.09), there is no significant connection between the target value and the sentiment feature, and the average sentence length is 12.

SemEval-2021 Task 7: The dataset contains 8000 training sets, 1000 validation sets, and 1000 test sets with an average length of 24.9. They collected 10,000 texts from Twitter and the Kaggle Short Jokes dataset, and had each annotated for humor and offense by 20 annotators aged 18–70.

The data distribution of the three datasets is shown in Table 1 below.

Evaluation indicators: because the humor recognition in this paper is only to determine whether the text is humorous or not, which is essentially a binary classification task, this paper uses evaluation metrics that are widely applied in classification tasks: accuracy (Acc), precision (*P*), completeness (*R*), and F-measure (F1). The datasets mentioned above are divided into training set, validation set, and test set according to the ratio of 8:1:1.

(17) $$P=TP/\left(TP+FP\right)$$

(18) $$R=TP/\left(TP+FN\right)$$

(19) $$F1=2*P*R/\left(R+P\right)$$

(20) $$Acc=\left(TP+TN\right)/\left(TP+FP+TN+FN\right)$$

Among them, TP (true positive) is a correct positive, indicating that the prediction is positive, and the prediction is correct, so it is actually a positive example. FP (False positive) is a false positive, which means that the prediction is positive, the prediction is wrong, and it is actually a negative case. FN (false negative) is a false negative, which means that the prediction is negative, the prediction is wrong, and it is actually a positive example.

### 5.2. Baselines

CNN + HN + F [41]: this method applies CNN with increasing filter size and highway layer [42] for humor recognition.

MAIS [33]: this method employs humorous snippet embeddings and contextual semantic inconsistency features to identify humor.

ABML [10]: The model unifies the two highly pertinent tasks, including the humor recognition and pun detection. In the ABML model, they design a co-encoder module to capture the common features between the two tasks by weight sharing. Apart from the co-encoder module, they also design two private encoder modules for the two tasks, respectively. The private encoder module is used to capture the private semantic feature of the two tasks.

BERT: Google released BERT in 2018 and successfully achieved the results of State Of The Art in 11 NLP tasks, winning praise from the natural language processing community. It is one of the current state-of-the-art natural language processing pretraining models.

XLNet [43]: Following BERT, Google and CMU jointly launched an improved version of BERT, XLNet, which optimized the shortcomings of BERT and achieved State Of The Art results in 18 tasks, especially in the field of text classification.

### 5.3. Experimental Settings

The model in this paper runs on a TITAN RTX GPU. The data of SemEval-2021 Task 7 has a fixed length of 64 after RoBERTa marking, and the length of Pun and 200K-Oneliners is 32. Similarly, the data for SemEval-2021 Task 7 has a fixed sequence length of 64 in the GloVe embedding, and a length of 32 for Pun and 200K-Oneliners. The length of phonetic embedding is fixed at 200. The hidden size of LSTM is 128, and the output channel size of the convolutional layer is 64. In GloVe word embedding, words that are not in the word list are always replaced by “<unk>”, and sequences that do not reach a fixed length are filled with “<pad>”. Stack transformer encoder self-attentive header is set to four and the number of layers is four. For the ambiguity embedding, the ambiguity level is initialized using vectors and the dimension is chosen to be four. ReLU is chosen as the activation function, dropout is set to 0.1 in the prediction layer, and the softmax function is applied. The batch size is 128, the learning rate is lr = $5\times 10^{-6}$, and learning rate decay is applied. The cross-entropy loss function is used as the loss function, the Adam optimizer is used for optimization, and the training epoch is 10.

### 5.4. Results and Analysis

Three sets of experiments were performed, and the baseline for each set is constructed as the following:

1. CNN+HN+F: the model settings are set according to the original text, the convolutional neural network filter size is (5–7), and the number of highway layer layers is three.
2. MAIS: the model is designed according to the original text, the snippet embedding adopts BERT, the semantic embedding adopts GloVe word embedding, and the convolutional neural network is applied to obtain the snippet embedding.
3. ABML: The model is fine-tuned on the original basis according to the characteristics of the dataset, and the other two datasets are trained together with the pun dataset.
4. BERT and XLNet: it is very convenient to train the pretrained model by using the pretrained model provided by Hugging Face [44] and the interface provided by the transformers package, choosing the hidden vector as the pretrained model of 768.

#### 5.4.1. Results

The results of the model in the three datasets are shown in the following three tables (Table 2, Table 3 and Table 4).

**Figure 2:**
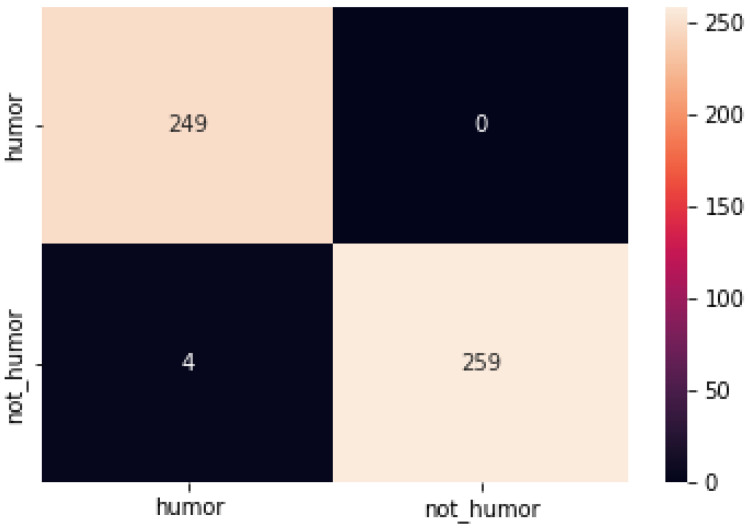
Confusion matrix of each model on Pun of the Day dataset.

**Figure 3:**
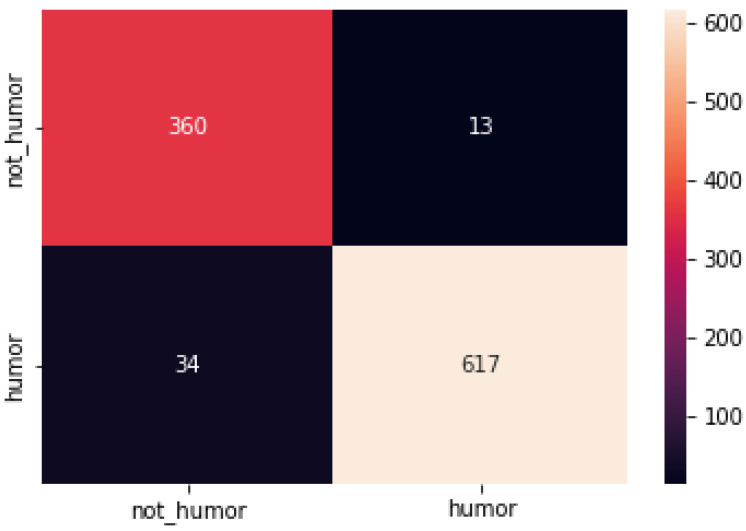
Confusion matrix of each model on model on SemEval-2021 Task 7 dataset.

**Figure 4:**
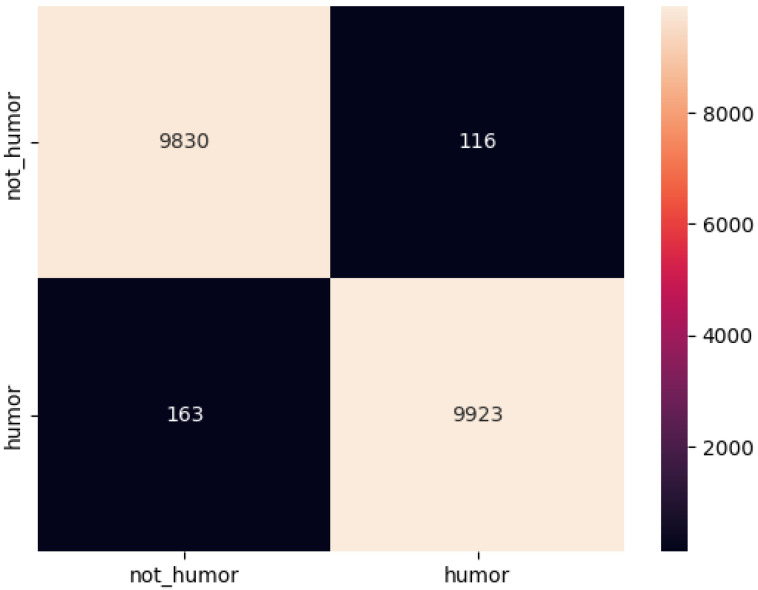
Confusion matrix of each model on model on 200k-Oneliners dataset.

The confusion matrix for the model on the three data sets is shown below (Figure 2, Figure 3 and Figure 4):

#### 5.4.2. Analysis

Table 2 below shows the results of the five baseline models and the model designed in this paper implemented on puns. From Table 2, it can be seen that the performance of the model designed in this paper is superior to all other models, proving the advanced nature of the proposed model in pun recognition.

For 200K-Oneliners, the large amount of data in this dataset led to good performance for each model, with BERT outperforming XLNet; although the difference in performance is small, the model in this paper is slightly better than XLNet.

The performance of each model in SemEval-2021 Task 7 is shown in Table 4. In this dataset, XLNet performs slightly better than BERT, but still not as well as the model in this paper, which again reflects that, in the field of humor recognition, extracting the deeper semantics of humor can better improve the performance of humor recognition.

By testing the model proposed in this paper and the most commonly applied language models on the above three datasets, it is concluded that in the field of humor recognition, the ability of humor recognization can be effectively improved by extracting semantic features based on humor theories, and the inconsistency of humor is better reflected not only between words but also between snippets.

## 6. Conclusions and Future Work

The main idea of this paper is to automatically recognize humor by extracting three types of features, namely, joke snippet inconsistency, phonetic features, and ambiguity, as well as sentence snippet embedding. These features are implemented based on humor theories. For each feature, this paper proposes relevant methods for extracting the corresponding features and combines these methods together to implement a compositive model. The model is validated on three publicly available saucy datasets and compared with the most popularly applied language models. Secondly, using the most commonly used pretrained language model word embedding can capture the semantics, proving that high-dimensional word vectors can express deeper lexical information, which is helpful for humor recognition.

However, this method still has some shortcomings; for example, the extraction method of speech features is still not comprehensive enough. Is there a better method? There is also no unified dataset standard for researchers to use. These shortcomings are to be corrected in future research.

In the future, we will continue to explore more and more effective features about humor based on humor theories and apply them to deep learning humor recognition, such as syntactic features, lexical features of jokes, etc., which are worthy research directions.

## Figures and Tables

**Table 1:** Sample distribution of each dataset.

Data	Positive	Negative
200K-Oneliners	100k	100k
SemEval-2021 Task 7	6179	3821
Pun-Of-The-Day	2403	2403

**Table 2:** Evaluation results of each model on Pun of the Day dataset.

Model	Acc	P	R	F1
CNN+HN+F(2018)	0.894	0.866	0.940	0.901
ABML(2021)	0.954	0.944	0.926	0.935
Bert(2018)	0.878	0.867	0.909	0.877
XLNet(2019)	0.988	0.986	0.984	0.992
MAIS(2021)	0.748	0.747	0.740	0.744
MLSN	**0.994**	**0.996**	**0.989**	**0.994**

**Table 3:** Evaluation results of each model on 200k-Oneliners dataset.

Model	Acc	P	R	F1
CNN+HN+F(2018)	0.943	0.955	0.930	0.943
ABML(2021)	0.955	0.957	0.954	0.955
Bert(2018)	0.985	0.987	0.983	0.085
XLNet(2019)	0.983	0.985	0.982	0.983
MAIS(2021)	0.959	0.961	0.958	0.960
MLSN	**0.986**	**0.988**	**0.984**	**0.986**

**Table 4:** Evaluation results of each model on SemEval-2021 Task 7 dataset.

Model	Acc	P	R	F1
CNN+HN+F(2018)	0.771	0.834	0.784	0.808
ABML(2021)	0.920	0.933	0.931	0.936
Bert(2018)	0.918	0.935	0.956	0.935
XLNet(2019)	0.921	0.944	0.927	0.935
MAIS(2021)	0.939	**0.958**	0.941	0.950
MLSN	**0.954**	0.945	**0.979**	**0.963**

## Data Availability

Not applicable.
